# Bis[*N*-(2-hydroxy­ethyl)-*N*-methyl­glycinato]copper(II)

**DOI:** 10.1107/S1600536808037793

**Published:** 2008-11-22

**Authors:** Elena A. Buvaylo, Volodymyr N. Kokozay, Olga Yu. Vassilyeva, Brian W. Skelton

**Affiliations:** a Department of Inorganic Chemistry, National Taras Shevchenko University, Volodymyrska Street 64, Kyiv 01033, Ukraine; b Chemistry, M313, School of Biomedical, Biomolecular and Chemical Sciences, University of Western Australia, 35 Stirling Highway, Crawley, WA 6009, Australia

## Abstract

The title compound, [Cu(C_5_H_10_NO_3_)_2_], was obtained unintentionally as the product of an attempted synthesis of a Cu/Cd mixed-metal mixed-anion complex using zerovalent copper, cadmium(II) oxide and two ammonium salts in the presence of 2-dimethyl­amino­ethanol in acetonitrile, in air. The mol­ecule is centrosymmetric with two monodeprotonated *N*-(2-hydroxy­ethyl)-*N*-methyl­glycines coordinated to the metal in a tridentate mode, giving a bicyclic chelate with two distorted five-membered rings. The Cu^II^ ion possesses a distorted octa­hedral geometry, with the N and the O atoms from the carboxyl­ate groups in the equatorial plane. In the crystal structure, inter­molecular O—H⋯O hydrogen-bonding inter­actions from the alkoxide functions of the ligand through the inversion centre form columns of mol­ecules propagated along the *a* axis.

## Related literature

For general background, see: Vinogradova *et al.* (2002 ▶, 2003 ▶); Farfán *et al.* (1987 ▶). For a related structure, see: Thakuria & Das (2007 ▶).
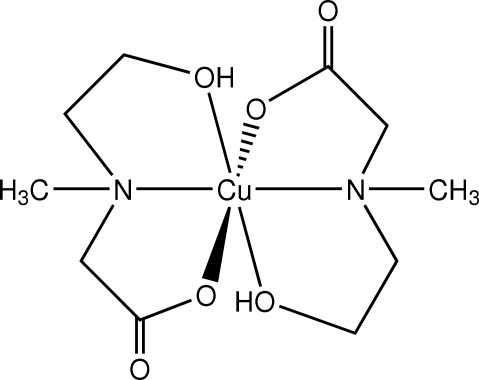


## Experimental

### 

#### Crystal data

[Cu(C_5_H_10_NO_3_)_2_]
*M* *_r_* = 327.82
Monoclinic, 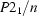

*a* = 6.6158 (9) Å
*b* = 6.7018 (9) Å
*c* = 14.950 (2) Å
β = 96.738 (3)°
*V* = 658.27 (15) Å^3^
*Z* = 2
Mo *K*α radiation
μ = 1.68 mm^−1^
*T* = 150 (2) K
0.15 × 0.13 × 0.10 mm

#### Data collection

Bruker SMART CCD diffractometer
Absorption correction: multi-scan (*SADABS*; Sheldrick, 1996 ▶) *T*_min_ = 0.74, *T*_max_ = 0.84
13931 measured reflections
3464 independent reflections
2539 reflections with *I* > 2σ(*I*)
*R*_int_ = 0.039

#### Refinement

*R*[*F* ^2^ > 2σ(*F* ^2^)] = 0.032
*wR*(*F* ^2^) = 0.085
*S* = 1.02
3464 reflections
93 parameters
H atoms treated by a mixture of independent and constrained refinement
Δρ_max_ = 0.79 e Å^−3^
Δρ_min_ = −0.32 e Å^−3^

### 

Data collection: *SMART* (Siemens, 1995 ▶); cell refinement: *SAINT* (Siemens, 1995 ▶); data reduction: *Xtal* (Hall *et al.*, 1995 ▶); program(s) used to solve structure: *Xtal*; program(s) used to refine structure: *SHELXL97* (Sheldrick, 2008 ▶); molecular graphics: *Xtal*; software used to prepare material for publication: *WinGX* (Farrugia, 1999 ▶).

## Supplementary Material

Crystal structure: contains datablocks I, global. DOI: 10.1107/S1600536808037793/hk2547sup1.cif

Structure factors: contains datablocks I. DOI: 10.1107/S1600536808037793/hk2547Isup2.hkl

Additional supplementary materials:  crystallographic information; 3D view; checkCIF report

## Figures and Tables

**Table 1:** Selected bond lengths (Å)

Cu—O1	2.4667 (9)
Cu—O2	1.9526 (8)
Cu—N1	2.0339 (9)

**Table 2:** Hydrogen-bond geometry (Å, °)

*D*—H⋯*A*	*D*—H	H⋯*A*	*D*⋯*A*	*D*—H⋯*A*
O1—H1⋯O3^ii^	0.880 (16)	1.84 (1)	2.713 (1)	172.1 (1)

Symmetry code: (ii) 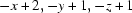.

## supplementary crystallographic information

### Comment

In our previous studies, Cd(II) salts were found to react with zerovalent copper and 2-dimethylaminoethanol in solution in air affording tetra-, penta- and hexanuclear assemblies that demonstrated significant structural flexibility of heterometallic cores made up of Cu, Cd, halide ions or acetate groups with O atoms from the aminoalcohol (Vinogradova *et al.*,  2002; Vinogradova *et al.*,  2003). As the reaction systems studied have been so productive in generating new Cd/Cu structures and in attempting to extend this family by employment of two different anions, we have examined the reaction of zerovalent copper, cadmium(II) oxide and two ammonium salts in the presence of the aminoalcohol in a non-aqueous solution, in air:

Cu^0^–CdO–NH_4_NCS–NH_4_I–2-dimethylaminoethanol–O_2_–CH_3_CN

However, an attempt to isolate a desired Cu/Cd mixed-metal mixed-anion complex failed. A by-product of the interaction was crystallographically identified that appeared to be a low-dimensional copper complex with new ligand HL, *N*-(2-hydroxyethyl)-*N*-methylglycine, formed *in situ*. The compound with HL in a monodeprotonated form, Cu(MeN(CH_2_CH_2_OH)(CH_2_COO))_2_, came as a surprise to us. To the best of our knowledge, neither the ligand nor its compounds with metals have been structurally characterized. The mechanism of the formation of the ligand is obscure, although it seemingly originates from 2-dimethylaminoethanol. It was reported that *N*-(2-hydroxyethyl)-*N*-alkylglycine derivatives could be conveniently prepared in high yields from *N*-alkylethanolamines and glyoxal (Farfán *et al.*,  1987). The authors noted that the method could not be used with N-unsubstituted ethanolamines, but fully N-substituted analogues such as 2-dimethylaminoethanol were not mentioned. We presume that in our case the conditions of the synthesis, namely the presence of zerovalent copper, could effect unknown organic reactions that led to the formation of HL.

In the title compound (Fig. 1), the two *L* molecules are coordinated to the metal in a tridentate mode giving a bicyclic chelate with two distorted five-membered rings, where the N and the O atoms from the carboxylic and the alkoxide functions are bonded to the metal ion. The copper(II) ion, that is bonded to the six atoms in an all-*trans* configuration, possesses a distorted octahedral geometry with N1 and O2 atoms from the carboxylate group in the equatorial plane (Table 1). The Cu—O1 bond in the axial position is substantially elongated (Table 1). The bond lengths (Table 1) are very similar to those observed in an analogous five-membered ring of bis(*N*,*N*-bis(2-Hydroxyethyl)glycinato)copper(II) (Thakuria & Das, 2007).

In the crystral structure, intermolecular O—H···O hydrogen-bonding (Table 2) interactions from the alkoxide functions of *L* through the inversion centre form columns of Cu*L*_2_ molecules propagated along the *a* axis (Fig. 2). The Cu···Cu separations in the crystal are larger than 6.6 Å.

### Experimental

For the preparation of the title compound, copper powder (0.16 g, 2.5 mmol), CdO (0.32 g, 2.5 mmol), NH_4_SCN (0.38g, 5 mmol), NH_4_I (0.72 g, 5 mmol), acetonitrile (25 ml) and 2-dimethylaminoethanol (2 ml) were heated to 323-333 K and stirred magnetically for 8 h, until total dissolution of the copper was observed. A fine green powder that precipitated immediately after cooling of the resulting solution was filtered off. The transparent blue solution was allowed to stand at room temperature and blue microcrystals suitable for X-ray analysis precipitated within 1 d. They were collected by filter-suction, washed with dry Pr^i^OH and finally dried *in vacuo* at room temperature (yield; 0.42 g). The crystals showed analytical data accounting for the presence of both Cu and Cd in the approximately 3Cu:Cd stoichiometry. However, the sample must has been a mixture as the X-ray structure investigation on a separate single-crystal conclusively established the identity of the compound as Cu(MeN(CH_2_CH_2_OH)(CH_2_COO))_2_. We were not able to separate this mixture by manual separation due to the visual uniformity of the crystals.

### Refinement

H1 atom (for OH) was located in difference synthesis and refined isotropically [O—H = 0.880 (16) Å and U_iso_(H) = 0.023 (4) Å^2^]. The remaining H atoms were positioned geometrically, with C—H = 0.99 and 0.98 Å for methylene and methyl H, respectively, and constrained to ride on their parent atoms with U_iso_(H) = xU_eq_(C), where x = 1.2 for methylene H and x = 1.5 for methyl H atoms.

### Crystal data

**Table:**

[Cu(C_5_H_10_NO_3_)_2_]	*F*(000) = 342
*M**_r_* = 327.82	*D*_x_ = 1.654 Mg m^−^^3^
Monoclinic, *P*2_1_/*n*	Mo *K*α radiation, λ = 0.71073 Å
Hall symbol: -P 2yn	Cell parameters from 3730 reflections
*a* = 6.6158 (9) Å	θ = 2.7–37.4°
*b* = 6.7018 (9) Å	µ = 1.68 mm^−^^1^
*c* = 14.950 (2) Å	*T* = 150 K
β = 96.738 (3)°	Prism, blue
*V* = 658.27 (15) Å^3^	0.15 × 0.13 × 0.10 mm
*Z* = 2	

### Data collection

**Table:**

Bruker SMART CCD diffractometer	2539 reflections with *I* > 2σ(*I*)
graphite	*R*_int_ = 0.039
ω scans	θ_max_ = 37.6°, θ_min_ = 2.7°
Absorption correction: multi-scan (*SADABS*; Sheldrick, 1996)	*h* = −11→11
*T*_min_ = 0.74, *T*_max_ = 0.84	*k* = −11→11
13931 measured reflections	*l* = −25→25
3464 independent reflections	

### Refinement

**Table:**

Refinement on *F*^2^	Primary atom site location: structure-invariant direct methods
Least-squares matrix: full	Secondary atom site location: difference Fourier map
*R*[*F*^2^ > 2σ(*F*^2^)] = 0.032	Hydrogen site location: inferred from neighbouring sites
*wR*(*F*^2^) = 0.085	H atoms treated by a mixture of independent and constrained refinement
*S* = 1.02	*w* = 1/[σ^2^(*F*_o_^2^) + (0.0446*P*)^2^] where *P* = (*F*_o_^2^ + 2*F*_c_^2^)/3
3464 reflections	(Δ/σ)_max_ = 0.046
93 parameters	Δρ_max_ = 0.79 e Å^−^^3^
0 restraints	Δρ_min_ = −0.32 e Å^−^^3^

### Special details

**Table:**

Geometry. All e.s.d.'s (except the e.s.d. in the dihedral angle between two l.s. planes) are estimated using the full covariance matrix. The cell e.s.d.'s are taken into account individually in the estimation of e.s.d.'s in distances, angles and torsion angles; correlations between e.s.d.'s in cell parameters are only used when they are defined by crystal symmetry. An approximate (isotropic) treatment of cell e.s.d.'s is used for estimating e.s.d.'s involving l.s. planes.
Refinement. Refinement of *F*^2^ against ALL reflections. The weighted *R*-factor *wR* and goodness of fit *S* are based on *F*^2^, conventional *R*-factors *R* are based on *F*, with *F* set to zero for negative *F*^2^. The threshold expression of *F*^2^ > σ(*F*^2^) is used only for calculating *R*-factors(gt) *etc*. and is not relevant to the choice of reflections for refinement. *R*-factors based on *F*^2^ are statistically about twice as large as those based on *F*, and *R*- factors based on ALL data will be even larger.

### Fractional atomic coordinates and isotropic or equivalent isotropic displacement parameters (Å^2^)

**Table:**

	*x*	*y*	*z*	*U*_iso_*/*U*_eq_	
Cu	0.5	0.5	0.5	0.01552 (6)	
O1	0.73510 (13)	0.22020 (14)	0.54077 (6)	0.02229 (17)	
H1	0.850 (2)	0.236 (2)	0.5761 (11)	0.023 (4)*	
O2	0.73795 (12)	0.64295 (13)	0.46744 (5)	0.01892 (16)	
O3	0.91657 (13)	0.69293 (14)	0.35177 (6)	0.02203 (17)	
N1	0.49180 (14)	0.36667 (14)	0.37742 (6)	0.01627 (17)	
C1	0.28504 (18)	0.3436 (2)	0.32884 (8)	0.0230 (2)	
H1A	0.215	0.4727	0.3262	0.035*	
H1B	0.2082	0.247	0.3606	0.035*	
H1C	0.295	0.296	0.2675	0.035*	
C2	0.58426 (18)	0.16401 (18)	0.39038 (8)	0.0204 (2)	
H2A	0.6117	0.1113	0.3311	0.024*	
H2B	0.485	0.0738	0.4143	0.024*	
C3	0.77970 (18)	0.16203 (19)	0.45363 (8)	0.0218 (2)	
H3A	0.8398	0.0266	0.4561	0.026*	
H3B	0.879	0.2557	0.432	0.026*	
C4	0.61451 (19)	0.49928 (18)	0.32557 (8)	0.0197 (2)	
H4A	0.5225	0.5922	0.289	0.024*	
H4B	0.6855	0.4174	0.2838	0.024*	
C5	0.77119 (16)	0.61937 (17)	0.38599 (8)	0.01686 (19)	

### Atomic displacement parameters (Å^2^)

**Table:**

	*U*^11^	*U*^22^	*U*^33^	*U*^12^	*U*^13^	*U*^23^
Cu	0.01503 (9)	0.01819 (9)	0.01393 (9)	−0.00255 (7)	0.00422 (6)	−0.00374 (7)
O1	0.0197 (4)	0.0263 (4)	0.0205 (4)	−0.0016 (3)	0.0010 (3)	−0.0004 (3)
O2	0.0186 (4)	0.0224 (4)	0.0164 (3)	−0.0043 (3)	0.0048 (3)	−0.0041 (3)
O3	0.0191 (4)	0.0280 (4)	0.0195 (4)	−0.0042 (3)	0.0048 (3)	0.0025 (3)
N1	0.0152 (4)	0.0188 (4)	0.0149 (4)	−0.0012 (3)	0.0020 (3)	−0.0025 (3)
C1	0.0172 (5)	0.0307 (6)	0.0206 (5)	−0.0023 (4)	−0.0004 (4)	−0.0065 (5)
C2	0.0225 (5)	0.0181 (5)	0.0208 (5)	−0.0010 (4)	0.0031 (4)	−0.0037 (4)
C3	0.0215 (5)	0.0202 (5)	0.0238 (5)	0.0029 (4)	0.0034 (4)	−0.0022 (4)
C4	0.0221 (5)	0.0227 (5)	0.0144 (4)	−0.0048 (4)	0.0028 (4)	−0.0003 (4)
C5	0.0163 (4)	0.0172 (5)	0.0172 (5)	0.0011 (4)	0.0026 (3)	0.0012 (4)

### Geometric parameters (Å, °)

**Table:**

Cu—O1^i^	2.4667 (9)	N1—C2	1.4928 (16)
Cu—O1	2.4667 (9)	C1—H1A	0.98
Cu—O2^i^	1.9526 (8)	C1—H1B	0.98
Cu—O2	1.9526 (8)	C1—H1C	0.98
Cu—N1^i^	2.0339 (9)	C2—C3	1.5097 (17)
Cu—N1	2.0339 (9)	C2—H2A	0.99
O1—C3	1.4235 (15)	C2—H2B	0.99
O1—H1	0.880 (16)	C3—H3A	0.99
O2—C5	1.2724 (14)	C3—H3B	0.99
O3—C5	1.2431 (14)	C4—C5	1.5227 (16)
N1—C1	1.4796 (14)	C4—H4A	0.99
N1—C4	1.4823 (15)	C4—H4B	0.99
			
O1—Cu—O2	86.05 (3)	N1—C2—C3	113.40 (10)
O1—Cu—N1	80.68 (3)	N1—C2—H2A	108.9
O2^i^—Cu—O2	180.00 (3)	C3—C2—H2A	108.9
O2^i^—Cu—N1^i^	85.87 (4)	N1—C2—H2B	108.9
O2—Cu—N1^i^	94.13 (4)	C3—C2—H2B	108.9
O2^i^—Cu—N1	94.13 (4)	H2A—C2—H2B	107.7
O2—Cu—N1	85.87 (4)	O1—C3—C2	108.45 (9)
N1^i^—Cu—N1	180	O1—C3—H3A	110
C3—O1—H1	109.0 (11)	C2—C3—H3A	110
C5—O2—Cu	114.36 (7)	O1—C3—H3B	110
C1—N1—C4	109.72 (9)	C2—C3—H3B	110
C1—N1—C2	108.06 (9)	H3A—C3—H3B	108.4
C4—N1—C2	111.85 (9)	N1—C4—C5	112.53 (9)
C1—N1—Cu	114.42 (7)	N1—C4—H4A	109.1
C4—N1—Cu	104.45 (7)	C5—C4—H4A	109.1
C2—N1—Cu	108.39 (7)	N1—C4—H4B	109.1
N1—C1—H1A	109.5	C5—C4—H4B	109.1
N1—C1—H1B	109.5	H4A—C4—H4B	107.8
H1A—C1—H1B	109.5	O3—C5—O2	125.00 (11)
N1—C1—H1C	109.5	O3—C5—C4	118.11 (10)
H1A—C1—H1C	109.5	O2—C5—C4	116.83 (10)
H1B—C1—H1C	109.5		

Symmetry codes: (i) −*x*+1, −*y*+1, −*z*+1.

### Hydrogen-bond geometry (Å, °)

**Table:**

*D*—H···*A*	*D*—H	H···*A*	*D*···*A*	*D*—H···*A*
O1—H1···O3^ii^	0.880 (16)	1.84 (1)	2.713 (1)	172.1 (1)

Symmetry codes: (ii) −*x*+2, −*y*+1, −*z*+1.

## References

[bb1] Farfán, N., Cuellar, L., Aceves, J. M. & Contreras, R. (1987). *Synthesis*, pp. 927–929.

[bb2] Farrugia, L. J. (1999). *J. Appl. Cryst.* **32**, 837–838.

[bb3] Hall, S. R., King, G. S. D. & Stewart, J. M. (1995). The *Xtal3*5 User’s Manual. University of Western Australia: Lamb, Perth.

[bb4] Sheldrick, G. M. (1996). *SADABS* University of Göttingen, Germany.

[bb5] Sheldrick, G. M. (2008). *Acta Cryst.* A**64**, 112–122. 10.1107/S0108767307043930 18156677

[bb6] Siemens (1995). *SMART* and *SAINT* Siemens Analytical X-ray Instruments Inc., Madison, Wisconsin, USA.

[bb7] Thakuria, H. & Das, G. (2007). *Polyhedron*, **26**, 149–153.

[bb8] Vinogradova, E. A., Kokozay, V. N., Vassilyeva, O. Yu. & Skelton, B. W. (2003). *Inorg. Chem. Commun* **6**, 82–85.

[bb9] Vinogradova, E. A., Vassilyeva, O. Yu., Kokozay, V. N., Skelton, B. W., Bjernemose, J. K. & Raithby, P. R. (2002). *J. Chem. Soc. Dalton Trans* pp. 4248–4252.

